# Improving the Health of Alaska Native People Through Use of a Policy Change Model and Capacity Building

**DOI:** 10.5888/pcd16.190077

**Published:** 2019-05-23

**Authors:** Diana Redwood, Kristen Mitchell-Box, Erin Peterson, Ellen Provost

**Affiliations:** 1 Alaska Native Epidemiology Center, Alaska Native Tribal Health Consortium, Anchorage, Alaska

## Abstract

Public health training often includes program and education development but not policy, systems, and environmental (PSE) strategies. The Alaska Native Tribal Health Consortium’s Good Health and Wellness in Indian Country program works to build tribal PSE change capacity. Trainings included community health assessment, facilitation and leadership engagement, policy and systems, and digital storytelling. From 2014 to 2017, 30 PSE changes were made: 3 tobacco-free healthcare organization policies; 2 tobacco-free tribal resolutions; 1 tobacco-free school district policy; 3 healthy food policies and environmental changes; 4 improvements in patient-provider communication; 13 prediabetes, obesity, and/or tobacco screening and referral policies; 3 improvements to health care facility signage; and 1 Baby-friendly Hospital application, protecting the health of 46,000 tribal community members. Targeted training and technical assistance moved tribal staff from a focus on direct services to population-based improvements. This increased self-efficacy may increase the sustainability of chronic disease public health efforts and improve tribal health.

**Summary**

**What is already known on this topic?**

Policy, systems, and environmental (PSE) improvements help support healthy behaviors. Despite the benefits of improving PSE factors, public health program staff are generally not trained in PSE-change skills.

**What is added by this report?**

Training and technical assistance activities increased Alaska tribal staff members’ ability to effect PSE change, resulting in 30 PSE improvements across the state.

**What are the implications for public health practice?**

Providing consistent, intensive technical assistance and focused trainings to increase self-efficacy were critical elements that helped staff move beyond traditional programmatic thinking and one-on-one clinical care. The use of digital storytelling was also a powerful tool that melded traditional Native storytelling with modern technology to enhance PSE change efforts.

## Background

Alaska Native people have significantly higher rates of chronic disease than non-Hispanic white people in the United States, and multiple health disparities exist between these 2 populations (1). Modifiable chronic disease risk factors, including unhealthy diet, physical inactivity, and tobacco use, are also common (1). Access to affordable groceries, fresh fruits and vegetables, and medical, dental, and behavioral health care services are limited in most rural or remote Alaska Native communities. Despite these challenges, Alaska Native people are strong and resilient, with traditions and values that facilitate a culture of health (2).

Changing the policy, systems, and environmental (PSE) factors that affect where people live, work, and play is increasingly being used nationally to support healthy behaviors and increase program sustainability after grant funding ends (3–5). Despite the benefits of improving PSE factors, public health program staff are generally not trained in PSE-change skills such as assessing community needs, developing policy, engaging stakeholders, countering resistance, navigating the policy landscape, working with nontraditional partners, or advocating for changes. These skills are important for ensuring that policy makers adopt, implement, and maintain PSE changes (6,7). In this article, we describe activities that increased tribal staff members’ ability to effect PSE change, outcomes of those efforts, and recommendations for others doing similar work.

## Good Health and Wellness in Indian Country Program

The Alaska Native Tribal Health Consortium (ANTHC) works in partnership with regional tribal health organizations (THOs) to promote health among Alaska Native people. THOs are responsible for providing care to the people in their region, and in most areas they are the only health care providers available. Services range from primary and emergency care services and behavioral and dental health care to health promotion programs. In 2014, ANTHC began a 5-year Good Health and Wellness in Indian Country (GHWIC) program to reduce rates of chronic diseases and their modifiable risk factors among Alaska Native people. The program focuses on supporting healthy behaviors through community-chosen and culturally adapted PSE approaches.

To achieve the goals of the program, ANTHC worked with 5 regional THOs in Alaska to increase access to traditional and healthy foods, increase physical activity, reduce tobacco use, improve health literacy, promote breastfeeding, and enhance chronic disease prevention and control. These THOs are responsible for providing care to 28% of the Alaska Native population living in 93 small communities (with an average population of ~440) (8).

## Capacity Building and the Policy Change Model

All regional THO GHWIC staff had extensive experience in providing clinical and health promotion services such as diabetes management but minimal or no experience in making PSE change. Many policy change models exist (9), but ANTHC chose the Rede Group’s policy change process model (Figure) to help systematically focus GHWIC training activities.

**Figure:**
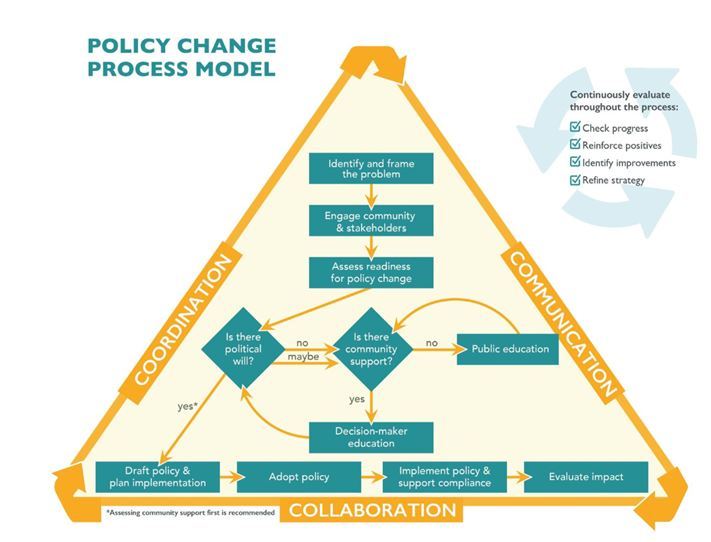
Policy change process model developed by the Rede Group (http://redegroup.co). The image is reproduced with permission from the Rede Group.

This model outlines 9 steps for policy change as well as a process for feedback loops and evaluation: it was general enough to allow for adaption to the tribal context of the GHWIC program. Regional THO GHWIC and ANTHC GHWIC staff attended 2-day to 4-day long trainings (15–25 participants per training). Trainees then used the PSE change skills at their THO and the tribal communities that their THO serves.

The first 3 steps of the policy change model focus on 1) identifying and framing the problem, 2) engaging the community and stakeholders, and 3) assessing readiness for policy change. Before the start of GHWIC, program staff members were familiar and comfortable with interacting with patient populations but less knowledgeable about identifying broader community health needs and priorities. To support partner sites and build capacity, ANTHC used the community health assessment training of the National Association of County & City Health Officials’ Mobilizing for Action through Planning and Partnerships. Participants learned a systematic approach for conducting community health assessments that resulted in new public health system assessments in THO communities and provided direction to focus PSE change efforts. The next 2 training sessions were facilitated by Technology of Participation (ToP) trainers (https://icausa.memberclicks.net). ToP provides training in structured participatory facilitation methods to help strengthen the capacities of organizations and communities. The first training session focused on how to effectively engage community members and stakeholders and build consensus despite competing priorities. The second training session covered strategic planning and how to build a 5-year program logic model to focus annual program activities.

Step 4 of the policy change model is to assess political will and community support. To build capacity in this area, the fourth training was led by the Midwest Academy, which provides civic engagement skills training to help achieve social, economic, and racial justice (www.midwestacademy.com). Participants learned approaches for identifying which constituencies and decision makers to engage to reach their goals. THO staff members use the training to learn how to think strategically, analyze power dynamics and political will of decision makers, build support within their community and organization, and strengthen their ability to make PSE improvements.

The focus of Step 5 in the policy change model is to assess the education and outreach needed for decision makers and for the public. Initially program staff were challenged about how to communicate the value of PSE changes. To help build these skills, and drawing on the rich history of Alaska Native storytelling, ANTHC hosted a digital storytelling workshop. Digital storytelling melds traditional storytelling techniques with technology to create short videos (3 minutes) that include audio, photographs, artwork, and music to tell a person’s story (10). During the workshop, THO staff members created digital stories to celebrate existing healthy policies, including a workplace that had breastfeeding policies to support new mothers. Other stories highlighted PSE changes needed to improve healthy behaviors, such as expanding health screenings and referrals and developing an organizational healthy food policy (anthctoday.org/epicenter/wsh.html).

THO staff members used digital stories for educating and engaging leadership and stakeholders within their organization. For example, one organization had passed a tobacco-free campus policy but had not yet implemented it. A THO tribal staff member created a digital story explaining how she and her children had to walk through cigarette smoke to get to their clinic appointments and how she hoped that tribal leadership would implement the policy as soon as possible. The digital story was shared with leadership, who set a date for policy implementation.

The last 4 steps of the policy change model are drafting policies and planning implementation, adopting policies, implementing policies and supporting compliance, and evaluating the impact of the policy change. For these 4 steps, the ANTHC GHWIC program provided ongoing intensive technical assistance and training sessions to regional THOs. This assistance and training covered such topics as health data provision, survey creation and analysis, focus group guide development, and draft policy language and editorial assistance, and it included an organizational health literacy assessment tool and other tools for assessing baseline policies and systems. More than 400 requests for technical assistance were completed in the first 3 years of the program alone.

## Building Public Health Capacity to Implement PSE Changes Among Alaska Native People

The skill and ability among THO staff members in Alaska to navigate the PSE change process within their organizations and communities has increased greatly since the start of the program in 2014. Across training opportunities, program staff members reported that these opportunities helped enhance their ability to identify, implement, and promote the use of PSE changes to improve population health. GHWIC staff members noted that the training on advocating for change, countering resistance, and engaging with leadership helped to strengthen their efforts to promote PSE changes more than traditional public health approaches that focus primarily on awareness building and education.

As a result of program efforts, 30 PSE changes were made. These changes included a tobacco-free policy at a health care organization (n = 3); a tobacco-free tribal resolution (n = 2); a tobacco-free policy in a school district (n = 1); a healthy food policy and environmental changes (n = 3); changes in patient–provider communication (n = 4); screening and referral policies for prediabetes, obesity, and/or tobacco (n = 13); changes in health care facility signage (n = 3); and an application for a Baby-Friendly Hospital designation (n = 1). These policies now protect the health of more than 46,000 tribal members and THO employees.

## Implications for Public Health Practice

Program staff members emphasized the importance of staying organized, having a multidisciplinary steering group, and including important stakeholders in the PSE change process, although some PSE changes took longer than expected because of external factors (eg, board priorities, changes in electronic record systems). Tribal leaders felt that the PSE changes made by the program had immediate positive impact on their organizations, including reducing tobacco use on the campuses of health care facilities, improving clinical quality measures, and serving as an “overall cultural change and shift.” Leadership noted the importance of using a strategic approach to introduce new policies and spending time gaining support.

The ANTHC GHWIC program activities have made significant PSE contributions to the health of Alaska tribal communities. These successes are all the more remarkable for occurring in small organizations providing multiple health services to remote tribal communities. Providing consistent, intensive technical assistance and focused trainings to increase self-efficacy were critical elements that helped staff move beyond traditional programmatic thinking and one-on-one clinical care. We recommend similar skill building and technical assistance for other tribal or public health organizations seeking to do PSE change work. The use of digital storytelling was also a powerful tool that melded traditional Native storytelling with modern technology to enhance PSE change efforts. PSE changes are often complex, involve multiple levels of an organization or community, and take more time to establish than traditional programs. Building these changes into organization norms and culture may substantially increase the sustainability of chronic disease public health efforts, leading to improved tribal health in the future.
